# Microfluidic Preparation of Janus Microparticles With Temperature and pH Triggered Degradation Properties

**DOI:** 10.3389/fbioe.2021.756758

**Published:** 2021-09-09

**Authors:** Zi-Yi Feng, Tao-Tao Liu, Zhen-Tao Sang, Zhen-Sheng Lin, Xin Su, Xiao-Ting Sun, Hua-Zhe Yang, Ting Wang, Shu Guo

**Affiliations:** ^1^ Department of Plastic Surgery, The First Affiliated Hospital of China Medical University, Shenyang, China; ^2^ School of Intelligent Medicine, China Medical University, Shenyang, China; ^3^ School of Forensic Medicine, China Medical University, Shenyang, China

**Keywords:** microfluidics, phase separation, Janus particle, phase change material, degradation

## Abstract

Based on the phase separation phenomenon in micro-droplets, polymer-lipid Janus particles were prepared on a microfluidic flow focusing chip. Phase separation of droplets was caused by solvent volatilization and Janus morphology was formed under the action of interfacial tension. Because phase change from solid to liquid of the lipid hemisphere could be triggered by physiological temperature, the lipid hemisphere could be used for rapid release of drugs. While the polymer we selected was pH sensitive that the polymer hemisphere could degrade under acidic conditions, making it possible to release drugs in a specific pH environment, such as tumor tissues. Janus particles with different structures were obtained by changing the experimental conditions. To widen the application range of the particles, fatty alcohol and fatty acid-based phase change materials were also employed to prepare the particles, such as 1-tetradecanol, 1-hexadecanol and lauric acid. The melting points of these substances are higher than the physiological temperature, which can be applied in fever triggered drug release or in thermotherapy. The introduction of poly (lactic-co-glycolic acid) enabled the formation of multicompartment particles with three distinct materials. With different degradation properties of each compartment, the particles generated in this work may find applications in programmed and sequential drug release triggered by multiple stimuli.

## Introduction

Janus particles are a kind of colloidal materials with non-centrosymmetric characteristics in morphology, composition and properties. It was first proposed by De Gennes, a French scientist, when he won the Nobel Prize in 1991 (de Gennes, 1992). The word “Janus” originated from the ancient Roman double-faced God, a reflection of its asymmetry. In recent years, as typical multifunctional materials, Janus particles have emerged in the fields of chemistry, physics, life sciences and so on (Hu et al., 2012; Kaewsaneha et al., 2013). The morphology of Janus particles is diverse including dumbbell, rod, snowman or any of a variety of other shapes, in addition, Janus particles assembled from block polymers or small molecules can also be divided into dendritic, vesicular, and other structures. According to the composition of particles, Janus particles can be divided into polymer type, inorganic type and polymer inorganic hybrid type (Su et al., 2019). Janus particles with nano/micron size have not only complex morphology, but also possess the inherent optical, magnetic and electronic properties in each part (Jin and Gao, 2009). It is well recognized that properties and applications of Janus particles are highly dependent on their morphology and chemical composition. Janus particles with adjustable size, biocompatibility, and low toxicity are widely used in biomedical fields, especially in the fields related to living cell research and tumor inhibition, such as cell encapsulation (Zhao et al., 2009; Maeda et al., 2012), cell imaging (Yi et al., 2016; Li et al., 2019), drug delivery (Xuan et al., 2014; M. El-Sherbiny and Abbas, 2016; Feng et al., 2019; Severino et al., 2019) and so on. At present, Janus particles have covered the whole range from nano scale to micron scale. Micron or submicron Janus particles can be prepared by a series of methods, the conventional preparation methods of Janus particles mainly include emulsion method, interface protection method, phase separation method, surface nucleation method and self-assembly method (Du and O’Reilly, 2011). These methods usually have high preparation throughput, and can realize the preparation of nano-sized Janus particles, some of these technologies have successfully prepared small-size Janus particles (Walther and Müller, 2013). However, the inherent limitations of conventional preparation methods are that it is difficult to achieve accurate control of particle morphology and to prepare particles with complex structure. In addition, the monodispersity and reproducibility of the particle production are relatively poor (Ali et al., 2021).

In recent years, microfluidic technology has gradually become a research hotspot, it is reported to have substantial applications in biology and medical research, for it can integrate a variety of complex biological and chemical reactions on a tiny chip, which greatly saves resources and costs (Hansen and Quake, 2003). Microfluidic platform is robust in producing polymer-based Janus particles. The main methods include droplet-based method and flow-lithography method. Among them, the droplet based method uses the micro-droplets with asymmetric morphology formed by shear force, electric field and centrifugal force in microfluidic system as the templates of Janus particles, combined with appropriate curing methods (Yu et al., 2021). While the flow-lithography method is a micro projection technology based on photo-polymerization of multiphase laminar flow. The fluid in the microfluidic chip is exposed to an ultraviolet beam with preset shape, causing the irradiated fluid to partially polymerize according to the beam shape to obtain Janus particles. The whole preparation process can be completed with the help of a commercial fluorescence microscope (Chizari et al., 2020; Sun et al., 2014). Microfluidic method can endow Janus particles with complex structure (such as three-dimensional asymmetric structure) or sophisticated morphology (such as graphic coding morphology) and the control of particle size, which greatly expands the design and application scope of Janus particles (Shepherd et al., 2006). However, the materials used in Janus particle preparation by microfluidic method are limited to polymers, which greatly restricts their application (Shakeri et al., 2021). Especially in drug delivery, the integration of a variety of materials is desired to achieve different degradation characteristics in a single carrier, so as to realize programmed, sequential or triggered release of different drugs.

In the present work, we fabricated Janus particles with temperature and pH triggered degradation characteristics by integration of pH sensitive polymers and phase change lipids in a microfluidic device. In addition, fatty alcohols, and fatty acids were also employed to produce the particles in order to achieve more controllable temperature triggered degradation. The triple-phase structure was also explored by adding a third material in order to realize a more programmed degradation mode. These particles are expected to have application potential in personalized and programmed drug delivery.

## Material and Methods

### Reagents

Poly (butyl methacrylate-co-(2-dimethylaminoethyl) methacrylate-co-methyl methacrylate) (p (BMA-co-DAMA-co-MMA), Eudragit E100) was perchased from Shanghai Dexiang Medicine Tech. Co., Ltd. Poly (lactic-co-glycolic acid) (PLGA) (50:50, Mw 10,000) was purchased from Jinan Daigang Biomaterial Co., Ltd., China. Hydrogenated coconut glyceride lipid (HCG lipid, Softisan 100) was purchased from Sasol (China) Co., Ltd., Nanjing, China. Dimethyl carbonate (DMC, >98%) was purchased from TCI (Shanghai) Development Co., Ltd., China. Polyvinyl alcohol (PVA, Mw 1750 ± 50), 1-tetradecanol, 1-hexadecanol, lauric acid, sodium dodecyl sulfate (SDS), Span 80 and dichloromethane (>99.5%) were purchased from Sinopharm Chemical Reagent Corporation, Shanghai, China. EM 90 was purchased from Evonik Industries, Shanghai, China. All of the above reagents were used as received without further purification, and deionized water was used throughout the experiment.

### Preparation of Microfluidic Chip

**FIGURE 1:**
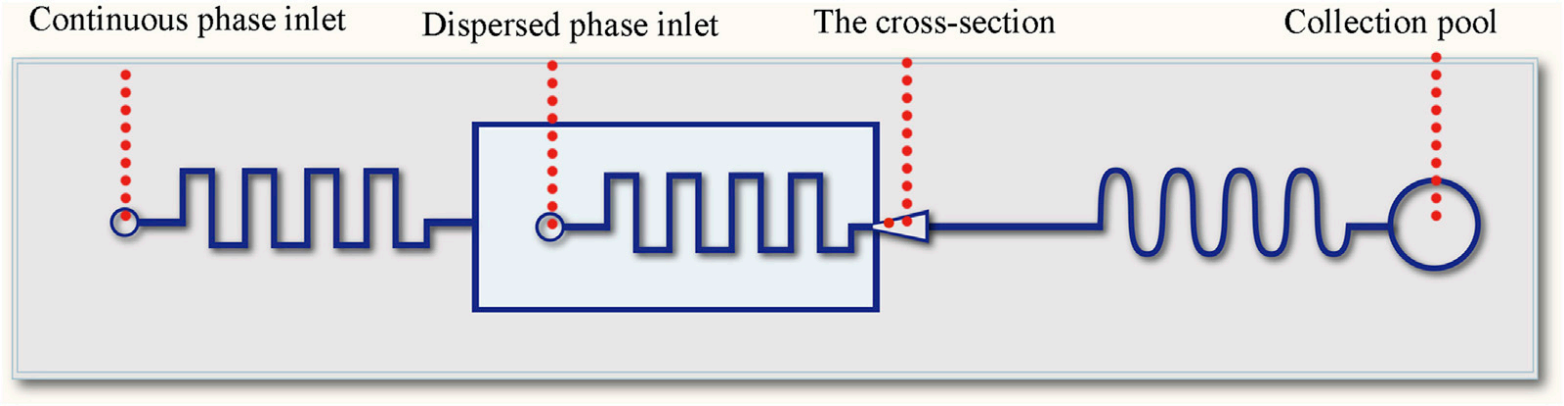
Schematic diagram of the microfluidic chip.

A flow-focusing sheath and winding channels were designed on a chip to fabricate the templet droplets, the schematic diagram is shown in Figure 1.

**FIGURE 2:**
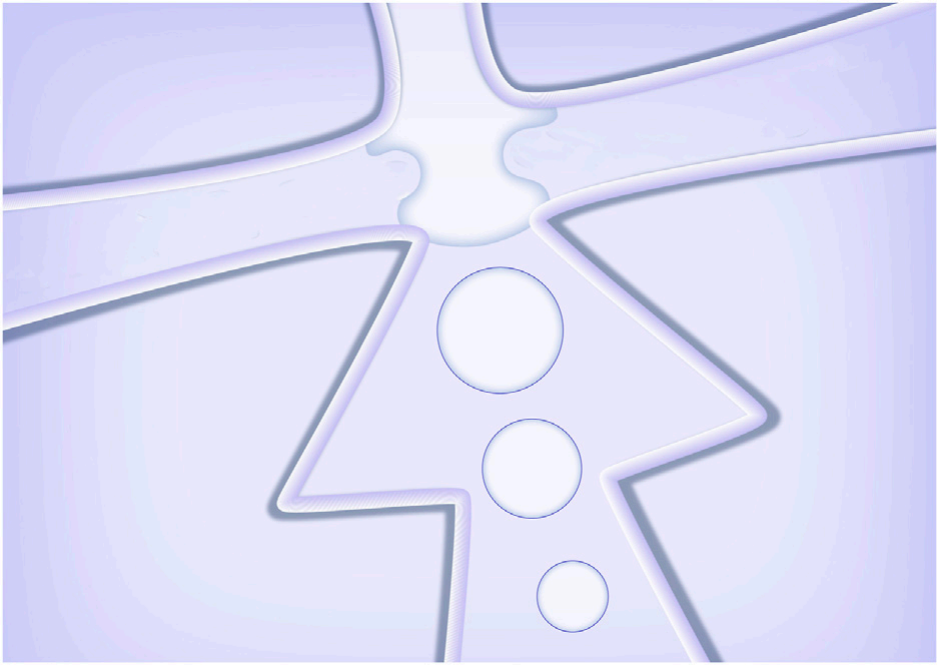
Schematic diagram of the droplets formed in the cross-section.

The whole device was made from polydimethylsiloxane (PDMS), consisting a channel layer and a substrate layer. The microfluidic channel was designed with flow focusing and winding structures. The flow focusing structure was used for the formation of oil-in-water droplets (Figure 2), and the downstream winding structure was used for the on-line solvent volatilization. The channel structure consists of a dispersed phase inlet, a continuous phase inlet and an outlet collection pool, and the phase separation process of droplets happened in the collection pool. The width of the winding channel for introducing the continuous and dispersed phase is 50 μm. The width of the main winding channel is 100 μm. The cross section of all channels is rectangular with a depth of 75 μm. The microfluidic chip was fabricated with the method of SoftLithography. Hydrophilic modification was carried out to produce oil-in-water droplets. 0.3 wt% PVA solution was applied to wash the channel for 0.5 h (flow rate 0.5 μl/min) to form a hydrophilic coating on the channel wall.

### Preparation of Solutions and Microparticles

A mixed solution of polymer and lipid (both concentrations were 5 mg/ml) in DMC with 0.5% (v/v) Span 80 was used as the dispersed phase. 0.3 wt% PVA solution with 0.1 wt% SDS was employed as the continuous phase. To prepare the particles, the solutions were injected into the microchip by syringe pumps (pump11 Elite, Harvard, United States) with glass syringes (Hamilton, Switzerland). The flow rates of dispersed phase and continuous phase were set at 1 μl/min and 2 μl/min, respectively. And the resulted particles were collected into a sample cell using a pipettor. The collected particles were afterwards placed in a refrigerator at 4°C for 5 min to cure the lipid, and then stirred under room temperature (around 25°C) for 2 h for complete removal of the solvent. The droplets and particles were observed by using a stereomicroscope with digital camera (MC-D500U, Phenix, China).

## Results

### Formation of Janus Droplets and Particles

Janus particles composed of materials with different physicochemical properties, have advantages when used as drug carriers in two main aspects: firstly, it is conducive to realize the segmented release control of drug loaded by particles, secondly, it is beneficial to realize the loading of different drugs. In this work, we focus on the p (BMA-co-DAMA-co-MMA) and HCG lipid which are approved by the U.S. Food and Drug Administration (FDA) to be used as drug additive (Mullard, 2020). p (BMA-co-DAMA-co-MMA) is widely used in gastric coating, enteric coating, sustained and controlled release coating, protective and isolation coating, sustained release matrix material and matrix adhesive material for transdermal drug delivery. p (BMA-co-DAMA-co-MMA) is formed by emulsion polymerization, it does not dissolve in neutral condition, but dissolves rapidly in the environment where the pH is lower than 5. HCG lipid has a melting point of 35 ± 1°C, and when it is used as a drug carrier, physiological temperature triggered phase transition and drug release will occur, thus it is often used in ointments, skin care product and drug microcarriers. Therefore, when the properties of these two materials are given to one Janus particle simultaneously, not only the segmented release control according to different temperatures and pH values can be realized, but also the sequential release when loading different drugs may be achieved.

**FIGURE 3:**
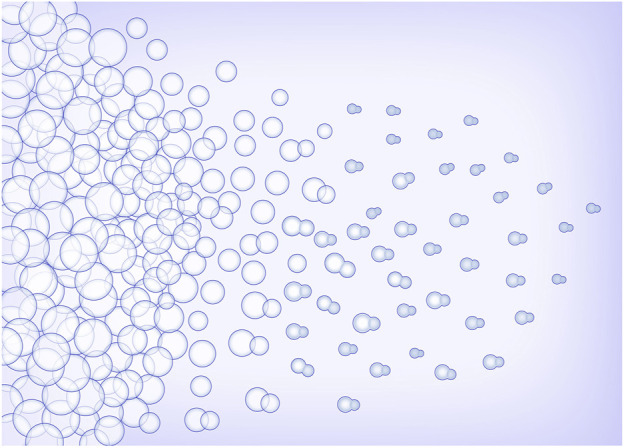
Schematic diagram of droplets phase separation.

However, it is difficult to prepare heterologous Janus particles by conventional methods, for the size and structure of the prepared particles are difficult to control. Microfluidic droplet technology can meet the demand of controllable preparation of heterologous Janus particles. Due to the difference in properties, the phase separation phenomenon in the droplet can be used to construct the Janus morphology. The typical process is that under the conditions of solvent volatilization, different components in droplets are separated spontaneously and gathered in different areas of the droplets respectively. We used the continuous phase to shear the dispersed phase to form the oil in water type droplets, and in the downstream a continuous winding channel was designed to allow for sufficient flow time for solvent evaporation. The permeability of PDMS allowed the solvent to evaporate into gas to avoid a large number or volume of bubbles. In general, the shape and aspect ratio of the channel had no significant effect on the droplet formation and phase separation. At the same time, due to the decrease of the flow rate (the flow rate of the dispersed phase would slow down when entering a wider channel from a narrow one), the droplets were easier to accumulate in the channel. However, if the flow time of the droplets in the chip was too short, the solvent evaporation of the droplet would be insufficient. Therefore, it is a necessity to maintain a reasonable width of the winding channel. After optimization, 100 μm was selected as the width of the winding channel. And after continuous test, 1 μl/min and 2 μl/min were chosen as the flow rates of the dispersed phase and the continuous phase, respectively. The process of droplet phase separation is shown in Figure 3.

**FIGURE 4:**
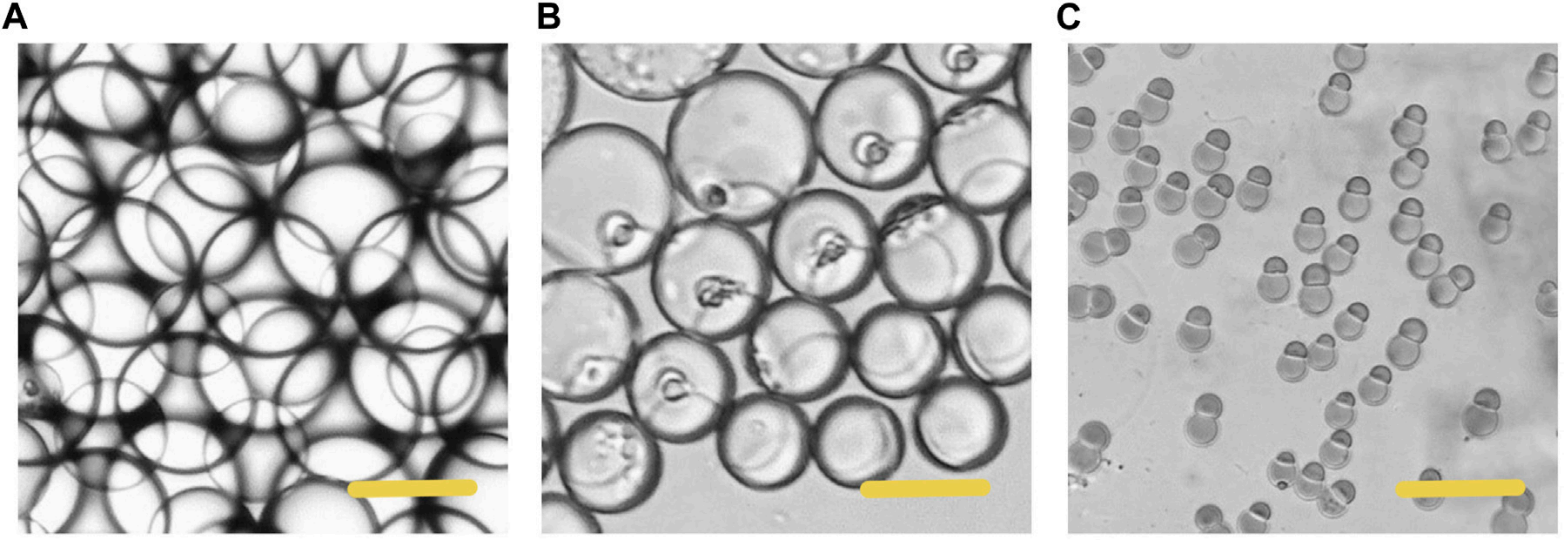
Microscopic images of the phase separation process of droplets **(A)** The initially formed droplet **(B)** The droplet size decreased with size decreased with solvent volatilization **(C)** The formation of the Janus particles. Scale bars represent 100 µm in all images.

In the process of droplet phase separation, the droplet size decreased with solvent volatilization, 100 μm (long diameter) at the beginning, while after 30 s in the pool, it dropped to 50 μm. After collecting the droplets and volatilizing the solvent, the short diameter of Janus particles was 20 μm and the long diameter was 40 μm. The droplet size decreased by 20% after another 2 h of the solvent evaporation. The results indicated that the efficiency of solvent evaporation was higher in the first 30 s in the collection pool. The rapid evaporation and phase separation can be attributed to the following aspects: firstly, the droplets formed in the microfluidic system are small in volume, usually in the order of pL or nL. Secondly, the design of the winding channel intensified the disturbance of the components in the droplet, thirdly, the selected solvent has good volatility. Figure 4 exhibits the whole process of the Janus particle formation. The droplets were formed at the flow focusing structure under the action of shear force. With solvent evaporating, the droplets shrunk and the concentrated contents started to separate from each other to minimize the Gibbs free energy (Min et al., 2016). When the droplet size decreased, the internal components of the droplet were stratified. This phenomenon was caused by the difference of affinity between polymer and lipid. With the solvent volatilizing, the two components in the droplet were gradually exposed to the water phase, and they had different solubility in the water phase, thus forming different solidification regions. In the final form of the Janus droplet, the interface between polymer and lipid was clear. What is more, the following off-chip quick-freezing and stirring were necessary to maintain the regular shape of the lipid compartments and to minimize the solvent residual.

### Degradation of the Lipid and Polymer Compartments

**FIGURE 5:**
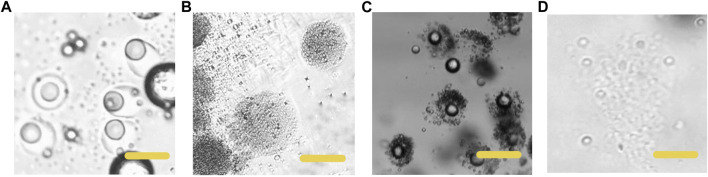
Microscopic images of the particles dissolving at different conditions **(A)** The HCG lipid melted in 37°C, PH 7.4 **(B)** The p (BMA-co-DAMA-co-MMA) dissolved when the environment pH was adjusted to 4,37°C **(C)** the p (BMA-co-DAMA-co-MMA) dissolved when the environment pH was 4,25°C **(D)** the HCG lipid melted when the temperature was raised to 37°C. Scale bars represent 100 µm in all images.

The lipid we selected would experience phase transition from solid to liquid under human body temperature. Therefore, we simulated the degradation process of particles at body temperature (37°C). Phase transition from solid to liquid of the lipid hemisphere is shown in Figure 5. The particles were heated at 37°C for 60 s, the lipid hemisphere began to melt and deform (Figure 5A). After incubation for 120 s, the lipid fraction was completely melted and adhered around the polymer hemisphere. However, from the aspect of appearance, the polymer hemisphere did not degrade. For the polymer hemisphere was able to dissolve in acid environment, we subsequently adjusted the environmental pH to 4, and observed that the remaining polymer dissolved completely (Figure 5B). On the contrary, when the pH was adjusted to 4, the appearance of lipid did not change significantly at room temperature (Figure 5C, Supplementary Video S1). After complete dissolution of the polymer, the left particles were heated to 37°C and maintained for a period of time, and the lipid then melted (Figure 5D, Supplementary Video S2).

This degradation mode plays an important role in many drugs delivery schemes. For example, in the therapy of some gastric diseases or tumor (Wang et al., 2021), local high-dose administration is a necessity to maintain the efficacy under acidic conditions. The release mode triggered by physiological temperature and environmental pH does not need the usage of photo-thermal materials. What is more, the whole preparation process of the particles is simple and fast. For all the materials we used are safe and non-toxic, which can be excreted from the body fluid circulation after administration. Due to the small mass and volume of particles, the effect of lipid on cardiovascular and cerebrovascular system can be ignored (Pardridge, 2012). Moreover, the dosage of drug carrier can be reduced by reasonably increasing the drug loading, so as to reduce the possible impact of drug carrier on human body. Although the lipid selected in our research has a low melting point, it can remain solid at normal room temperature. After being made into drug loaded particles, it can also be stored at a lower temperature. The time required for complete melting is enough to complete the delivery of particles to the therapeutic target by injection. Because the pH of tumor or some organs of the body is different from that of the surrounding (Ramsay and Carr, 2011), targeted drug delivery can be achieved in this mode.

### Control of Particle Phase Ratio

**FIGURE 6:**
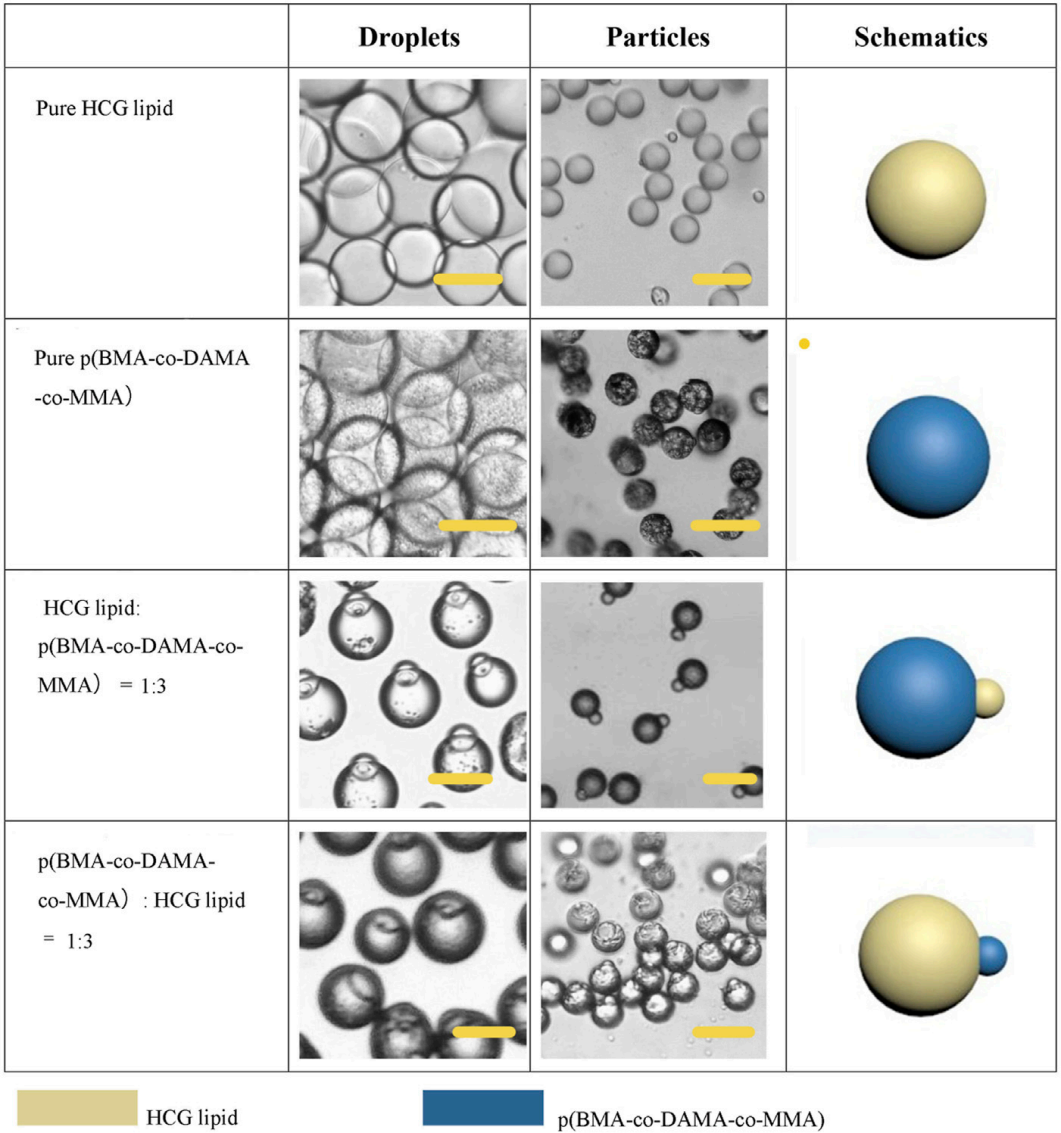
Schematic diagrams and microscopic images of droplets and particles with different phase ration. Scale bars represent 100 µm in all images.

Firstly, we explored the Janus particles and droplets formed by different proportions of lipid and polymer. Figure 6 shows the structure diagram and micrographs of droplets and particles with different compositions. When the particles were dispersed in water, they tended to be vertically arranged due to the low density of lipid, thus some of the particles in some photos do not show biphasic morphology. After phase separation, the proportion of two phases in the droplet was basically the same as that in the dispersed phase solution, and the particles could also maintain the present proportion of the two phases. Single phase polymer or lipid particles can be obtained by volatilization of solvent in droplets containing a single material. These particles also have high uniformity. Multiphase laminar flow can achieve the same goal, the volume ratio of different phases in the droplet is usually controlled by adjusting the flow rate ratio of different phases in laminar flow (Gao and Chen, 2008). However, it is difficult to accurately control the volume ratio of two phases and the mass ratio of two phases in solidified particles. Moreover, when the velocity difference between two phases is large, the phase with low velocity may not flow into the channel and form multiphase laminar flow with the other phase. In the method proposed here, the phase separation based preparation method does not need the formation of laminar flow. Only by fixing the component ratio of the solution, the volume ratio of the two phases in a droplet and a particle can be accurately controlled without adjusting the flow rate. Flexible control of phase ratio of Janus particles is quite essential when used as drug carriers and therapeutic factors. For example, when this kind of particle is used as a drug carrier, the phase ratio can be adjusted according to the actual application to regulate the release amount at different release stages or the loading ratio of two drugs.

### Structure Control

**FIGURE 7:**
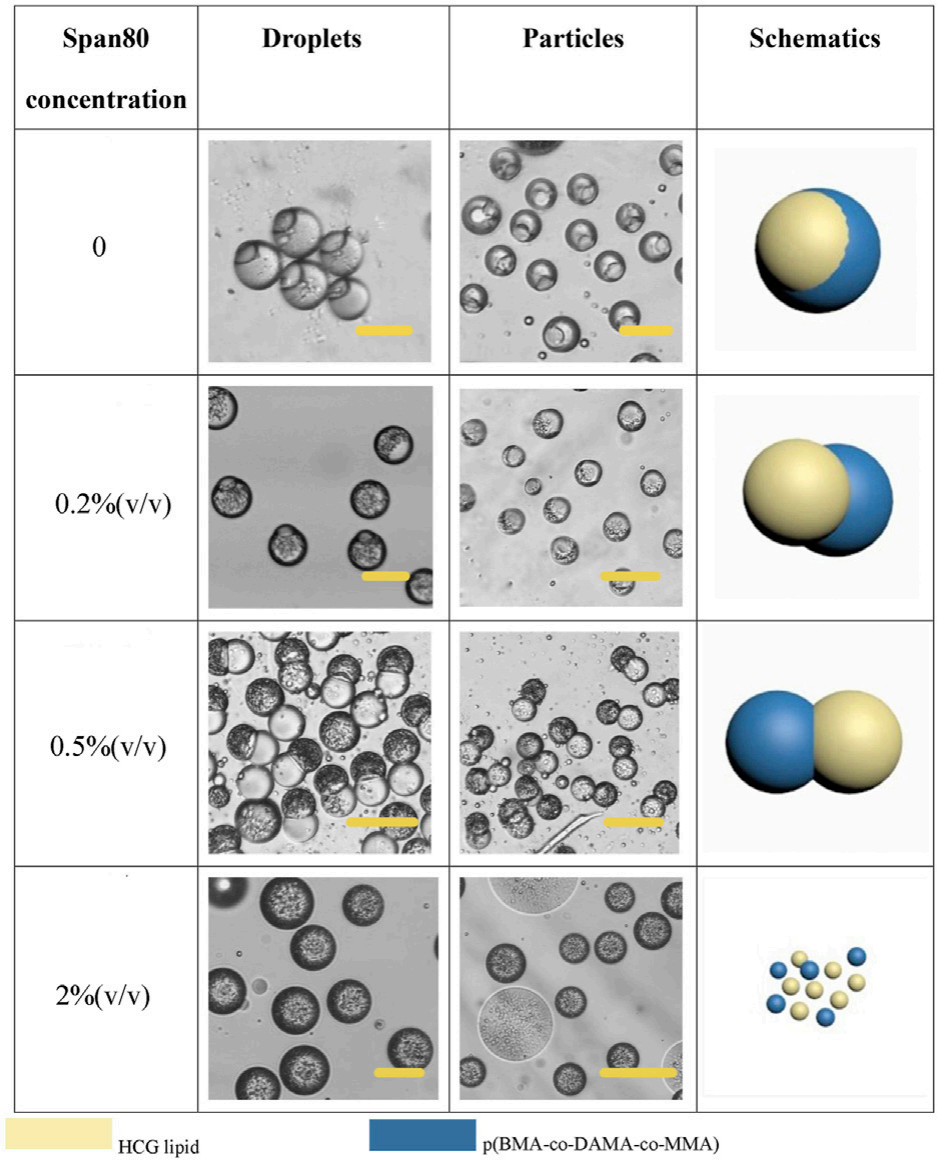
Schematic diagrams and microscopic images of droplets and particles with different Span 80 concentration Scale bars represent 100 µm in all images.

In addition to the phase ratio, the effect of other factors on the morphology of Janus particles were also explored. In fact, the final morphology of the droplet is determined by the interfacial tension between different phases, and the wettability between different phases is reflected by the spreading coefficient (*Si*) (Sun et al., 2019). Under our experimental conditions, when the concentration of Span 80 is 0.5% (v/v), the Janus (dumbbell) shape formed. As is well accepted that surfactant is pivotal to regulate the interfacial tension, we further investigated the role of Span 80 in this system. The concentration of Span 80 affected the degree of particle separation, as is shown in Figure 7. When the concentration of Span 80 was varied from 0 to 0.5% (v/v), the lipid and polymer hemispheres became apart from each other gradually. But when we increased the concentration to 2% (v/v), the law was broken. Janus particles were no longer formed. Lipids and polymers had no independent solidification areas. Instead, a whole droplet was wrapped with many small particles. With the evaporation of the solvent, the particles became unstable and broke up spontaneously. This phenomenon may attribute to the fact that high concentration of Span 80 surfactant led to tardy separation of the two phases, as well as a low interfacial tension between the organic phase and the aqueous phase, which resulted in an increased *Si* of the aqueous phase and therefore completely separated lipid and polymer.

**FIGURE 8:**
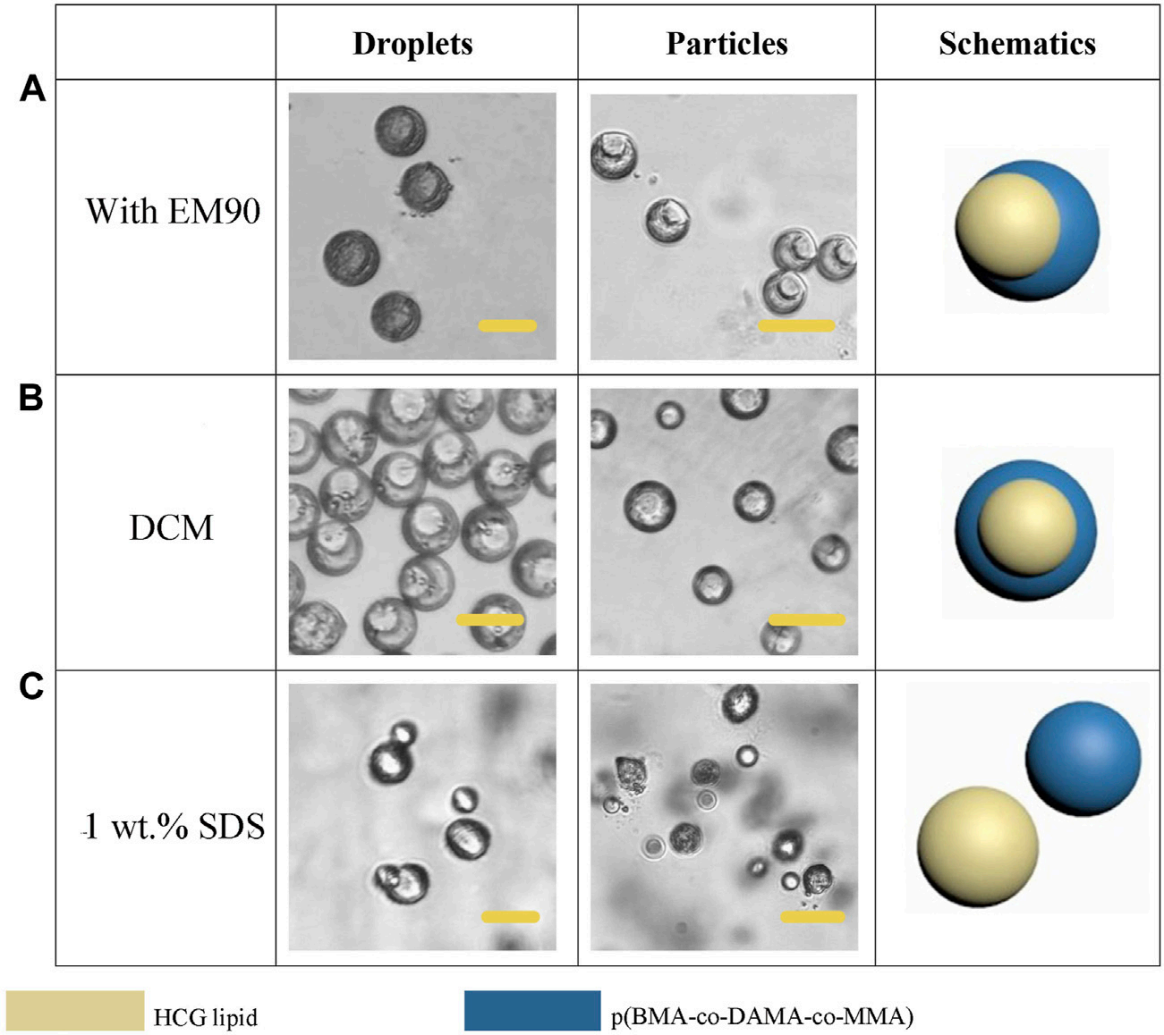
Schematic diagrams and microscopic images of droplets and particles prepared with EM90 as surfactant **(A)**, DCM as solvent **(B)** and increased SDS concentration **(C)**. Scale bars represent 100 µm in all images.

EM 90 was also tested as the surfactant in the organic phase, when 0.5% (v/v) EM 90 was applied, the particles turned into an eccentrically encapsulated core-shell structure with the lipid compartment as an inner core (Figure 8A). Solvent also has a significant impact on the particle morphology. When we used DCM as the solvent for dispersed phase, most of the lipid formed a complete sphere and gathered in the center of the polymer shell (Figure 8B). SDS was employed as an aqueous anionic surfactant, and increasing of the concentration of SDS in the aqueous phase to 1 wt.% would allow for the total separation of two compartments, for a higher SDS concentration led to restraint of the interfacial tension between the aqueous phase and the polymer/lipid phase, the *Si* of the aqueous phase turned to be more positive, leading to complete dewetting of the organic phases (Figure 8C). And the size and shape of particles may be further broadened by optimizing the experimental conditions.

### Exploration of Fatty Alcohol-and Fatty Acid-Based Phase Change Materials

**FIGURE 9:**
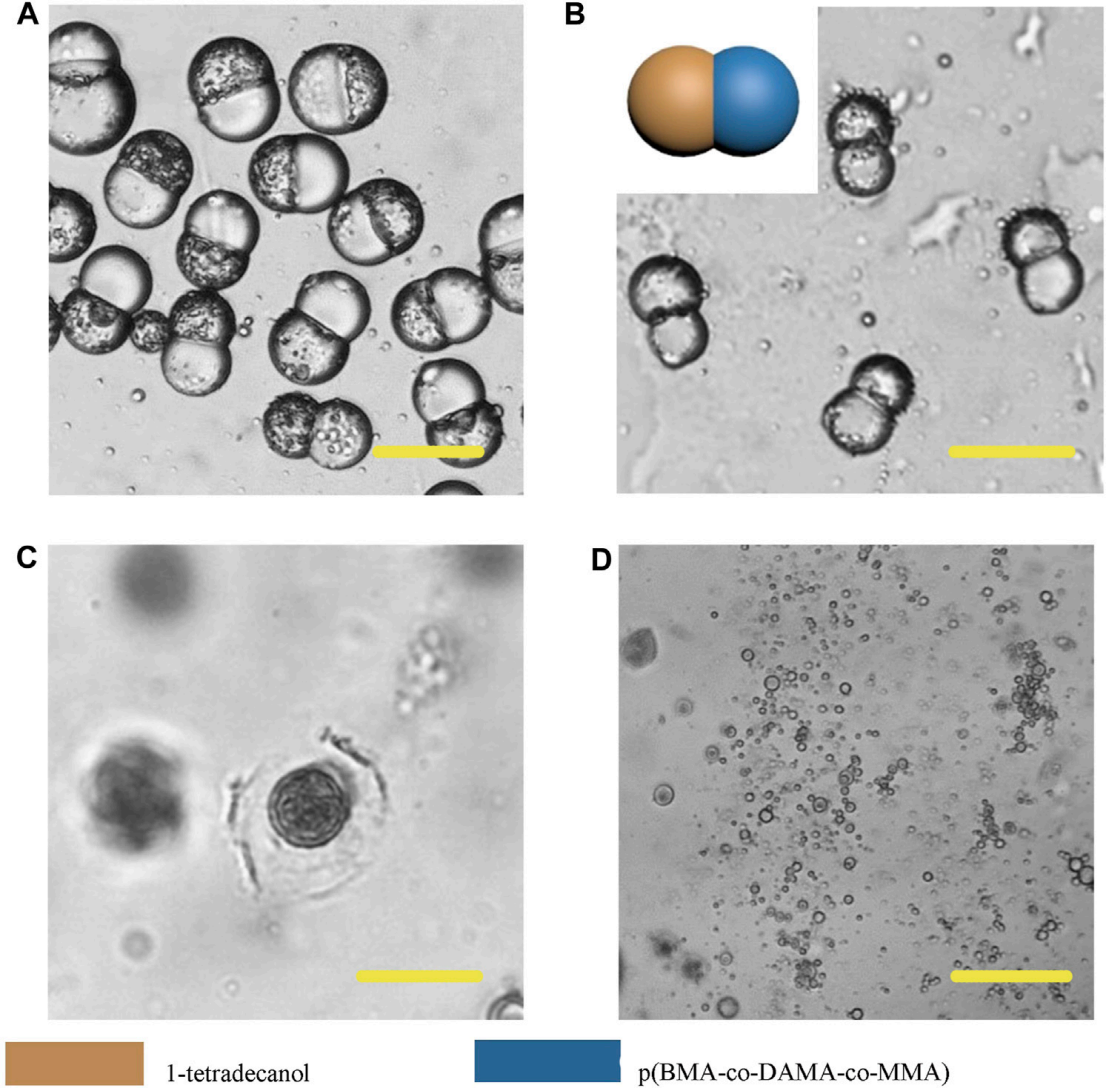
Microscopic images of droplets **(A)**, particles **(B)** composed of 1-tetradecanol and p (BMA-co-DAMA-co-MMA) **(C)** 1-tetradecanol region melted at 39°C **(D)** The p (BMA-co-DAMA-co-MMA) degraded in pH 4 environment Scale bars represent 100 µm in all images.

**FIGURE 10:**
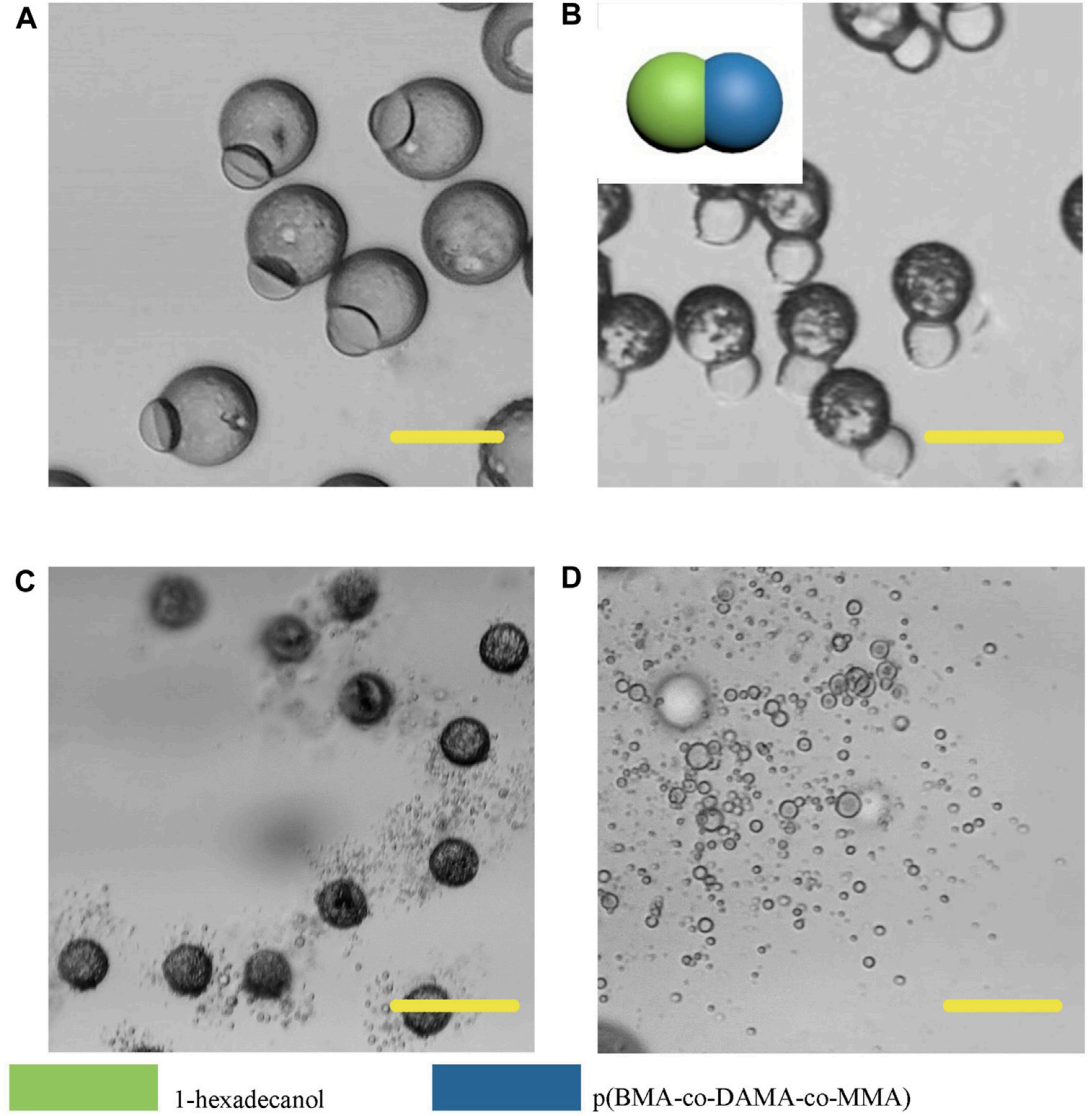
Microscopic images of droplets **(A)**, particles **(B)** composed 1-hexadecanol and p (BMA-co-DAMA-co-MMA) **(C)** 1-hexadecanol region melted at 49°C **(D)** The p (BMA-co-DAMA-co-MMA) degraded in pH 4 environment Scale bars represent 100 µm in all images.

In order to further broaden the application scenarios of Janus particles, we tested more phase change materials to combine with the polymer. The phase change material is a kind of thermosensitive material which can transform into a liquid phase when heated to melting points (Choi et al., 2010). And some fatty alcohols and fatty acids are typical phase change materials. Here, 1-tetradecanol (melting range 38 and 39°C), 1-hexadecanol (melting range 47–49°C) and lauric acid (melting point 49°C) were selected to serve as the temperature sensitive hemisphere in Janus particles. In the case of an elevated body temperature due to fever, inflammation, some other diseases or during thermal-therapies, the lipid compartments would melt and rapidly release the loaded drugs (Hyun et al., 2014). We adopted the same experimental conditions except for changing the phase change materials. The results showed that the 1-tetradecanol (Figure 9) and 1-hexadecanol (Figure 10) exhibited similar Janus (dumbbell) structure with the HCG lipid. The interface of the two phases is clear, where the dark rough hemisphere is polymer, and the white smooth hemisphere is lipid. The degradation of lipid and polymer hemispheres were further investigated, we heated the particles to 39 and 49°C, respectively. The lipid phase melted at the corresponding melting point, while the polymer phase did not show a significant change. Then we adjusted the environment pH of the particles to 4, Similarly, the polymer degraded rapidly and the entire hemisphere collapsed.

**FIGURE 11:**
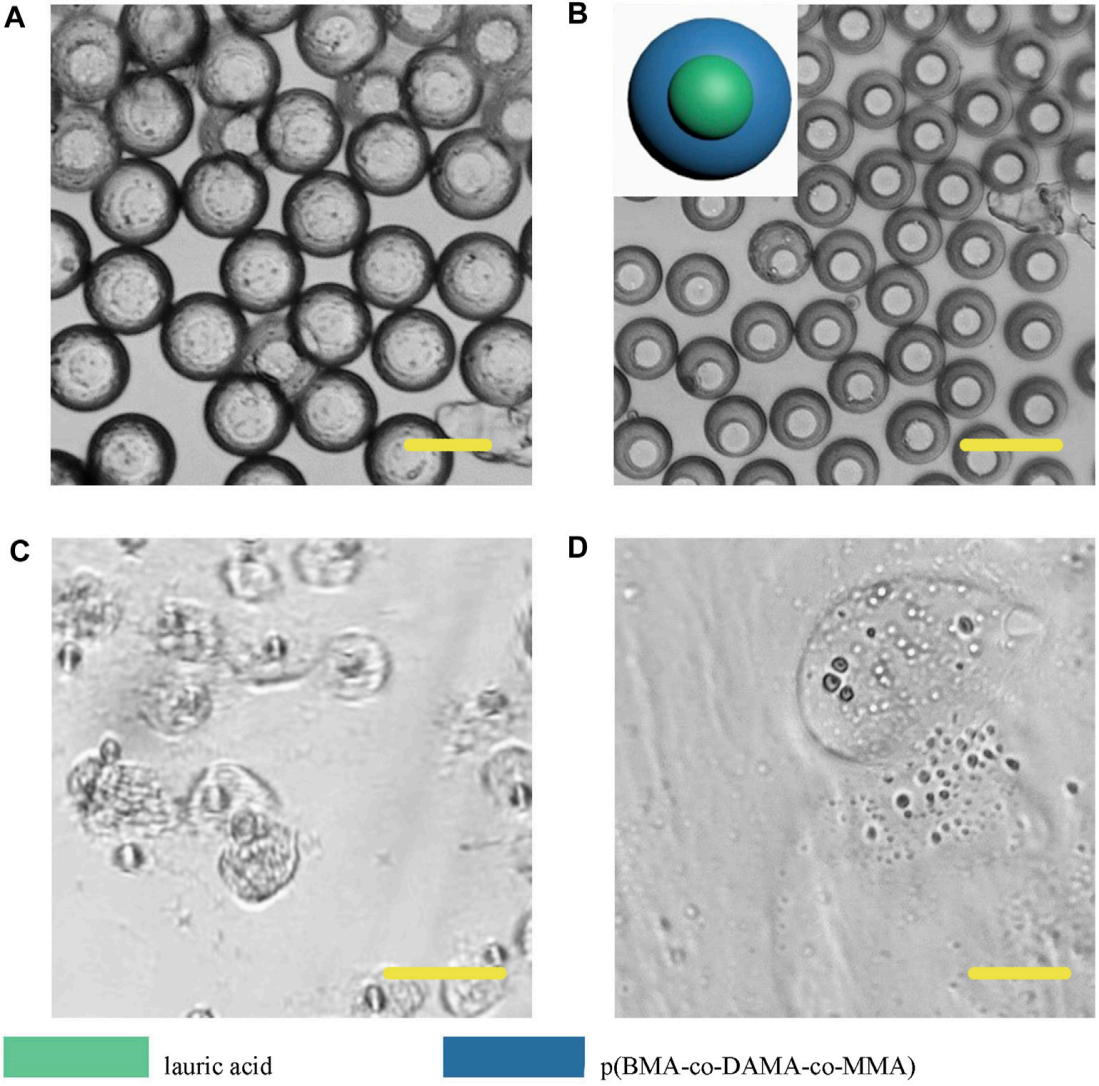
Microscopic images of droplets **(A)**, particles **(B)** composed of lauric acid and p (BMA-co-DAMA-co-MMA) **(C)** Lauric acid region melted at 49°C **(D)** The p (BMA-co-DAMA-co-MMA) degraded in pH 4 environment Scale bars represent 100 µm in all images.

However, different from the previous results, Janus particles made from lauric acid and polymer formed core-shell structure (Figure 11). When we heated the particle dispersing solution to 49°C, the particle surface did not appear any change, but when we adjusted the pH to 4, the particle shell degraded, thus we could infer that the outer layer was the p (BMA-co-DAMA-co-MMA) and the inner layer was the lauric acid. After the outer polymer was completely dissolved, we raised the temperature to 49°C, and the inner layer melted instantaneously. This result confirmed our hypothesis. The exploration of fatty alcohols and fatty acids has expanded the scope of materials that can be introduced to fabricate Janus particles in a microfluidic device, and has also increased more possibilities for further application.

### Preliminary Study on Multicompartment Microparticle Generation by Phase Separation

**FIGURE 12:**
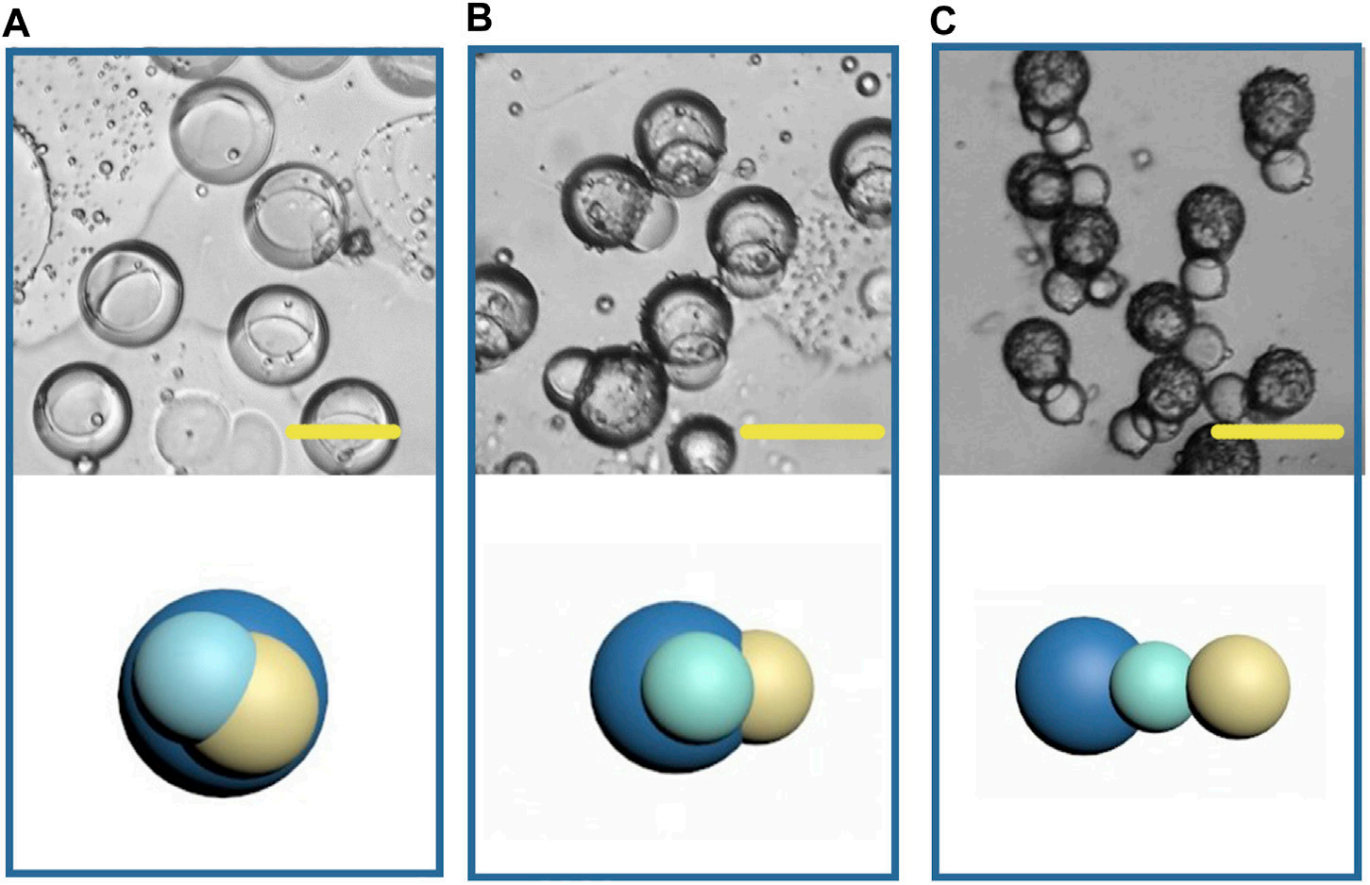
Microscopic images and the schematic diagrams of multicompartment droplets **(A)**, particles **(B, C)**. Scale bars represent 100 µm in all images.

In recent years, the fabrication of double-phasic particle by phase separation method has been gradually improved, but study on multicompartment particle fabrication by this method is rare. Based on the previous study of biphasic Janus particles, we tried to construct multicompartment particles to enrich the function of particles. We applied a third polymer, PLGA, due to its well acknowledged capability in providing sustained drug release. Some studies have indicated that PLGA took 35 days to completely degrade in phosphate buffered saline *in vitro* (Ding and Zhu, 2018). Therefore, the addition of PLGA in the whole system can achieve sustained release, temperature triggered release and pH triggered release with a same particle. The size of the newly formed droplets containing three components was 150 μm (long diameter), larger than that of the Janus droplets, and the structure of the triple droplets is shown in Figure 12A. As the solvent volatilized, the components in the droplet were gradually exposed to the water phase and because of their different solubility, different solidification regions formed, and then gradually formed multicompartment particles. In the newly formed particles, lipid located on one side, and the core-shell structure formed by the two polymers was on the other side (Figure 12B). When the phase separation process was complete, a final structure with three compartments linking together was observed (Figure 12C). This structure may be desired in programmed drug release, coding, self-assembly, and so on. And a series of triple structure may be fabricated through interfacial tension regulation in the future work.

## Discussion

Firstly, we prepared polymer-lipid Janus microparticles based on phase separation of micro-droplets. The microparticles have dumbbell like structure and clear two-phase interface. The lipid hemisphere made from HCG lipid can degrade at physiological temperature, while the polymer hemisphere made from p (BMA-co-DAMA-co-MMA) can degrade in acidic environment, which can meet the demand of drug release at a target site, such as tumor tissue. Moreover, the particles prepared in microfluidic system have high homogeneity and tunability of size and morphology, which makes it easier to achieve effective control of drug release when the particles are used as drug carriers. 1-tetradecanol, 1-hexadecanol and lauric acid were employed to mix with the polymer to produce Janus particles, with an aim to adjust the trigger temperature for degradation of the lipid hemispheres. The development of multicompartment structure by phase separation was also explored. PLGA was added on the basis of HCG lipid and p (BMA-co-DAMA-co-MMA) to achieve sustained drug release property. The triple particles possessed a linear structure with three compartments.

**FIGURE 13:**
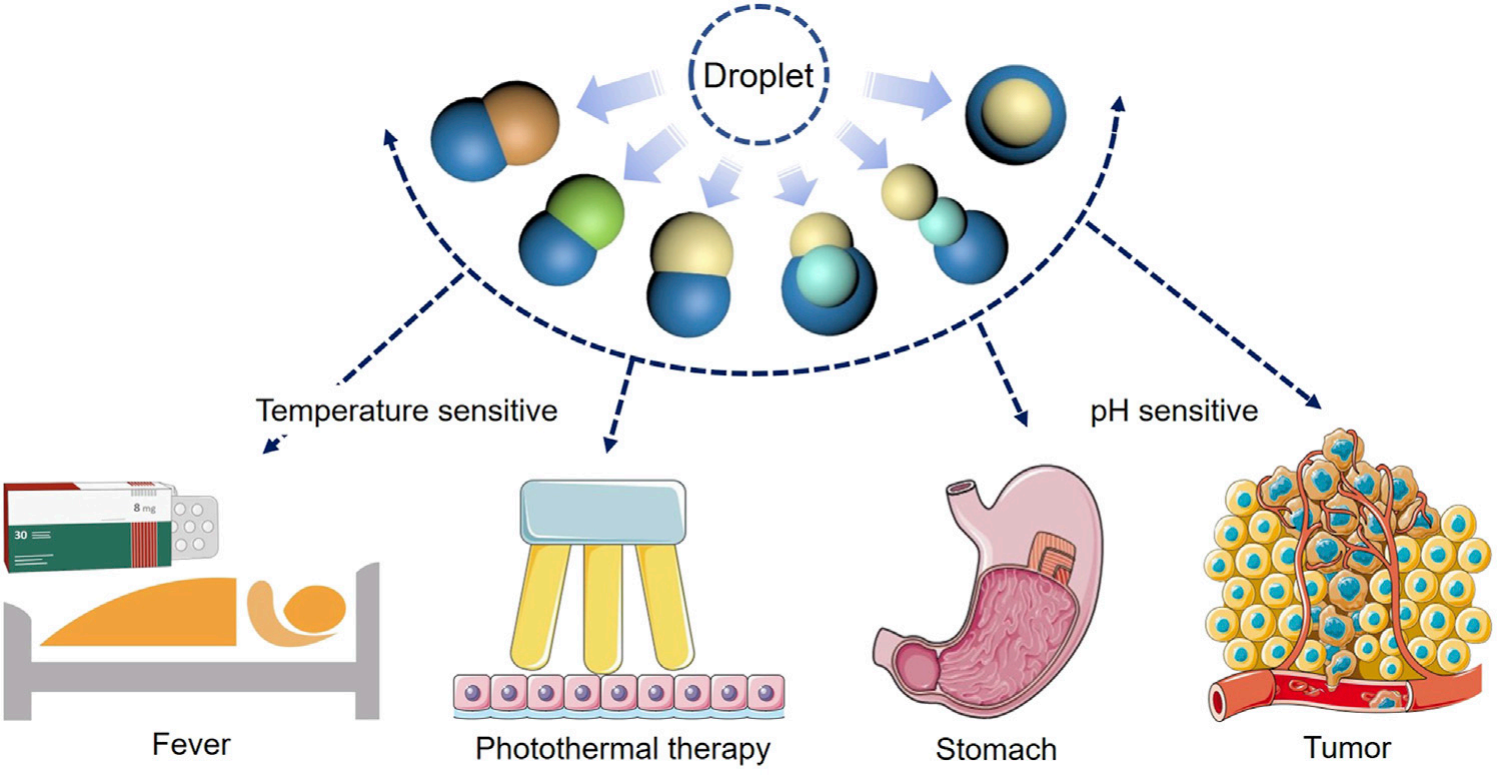
Schematic diagrams of the function of the Janus particles.

The microfluidic preparation method established in this work is simple and mild, and has universality in a variety of polymer and lipid materials, which provides a path for the preparation of functional heterologous Janus particles. In addition, due to the unique structure of Janus particles, different drugs or other cargoes can be simultaneously loaded in the particles. According to the practical or personalized demands, various types of drug loading particles can be prepared by regulating the particle structure to form a controlled release system with different combinations of synergistic drugs. Compared with using polymers to manufacture Janus particles, the most significant innovation of this work is that phase change materials (1-tetradecanol, 1-hexadecanol and lauric acid) were introduced to phase separation systems, which had different physical and chemical properties from polymers. Integration of polymer and phase change materials had given rise to one Janus particle with different degradation properties. And the two materials were sensitive to different stimuli, enabling triggered degradation of each compartment. The future work can be devoted to the achievement of more advanced phase separation systems and more complex structures, so as to realize programed degradation and drug release in many scenarios (Figure 13).

## Data Availability

The original contributions presented in the study are included in the article/Supplementary Material, further inquiries can be directed to the corresponding author.
